# Digital ≠ paperless: novel interfaces needed to address global health challenges

**DOI:** 10.1136/bmjgh-2021-005780

**Published:** 2021-04-20

**Authors:** Pratap Kumar, Stephen M Sammut, Jason J Madan, Sherri Bucher, Meghan Bruce Kumar

**Affiliations:** 1 Institute of Healthcare Management, Strathmore University Business School, Nairobi, Kenya; 2 Health-E-Net Limited, Nairobi, Kenya; 3 Health Care Management Department, University of Pennsylvania, Philadelphia, Pennsylvania, USA; 4 University of Warwick Warwick Medical School, Coventry, UK; 5 Department of Pediatrics, Indiana University School of Medicine, Indianapolis, Indiana, USA; 6 MARCH Centre, London School of Hygiene and Tropical Medicine, London, UK

**Keywords:** health systems, public health, health economics

**Summary box**

- Effective information systems do not always need to be accompanied by information technologies.
- Information technologies do not need to be ‘paperless’, and can benefit from the numerous advantages of paper-based information entry.
- Automated digitisation of paper-based information by taking a picture can deliver routine health information easily, accurately and at low cost.
- Hybrid, paper-digital systems could overcome common barriers to technology implementation and use - the need for infrastructure and repeated training - and help bridge current circumstances and ‘ideal’ information systems of the future.

## Introduction

Health information systems (HISs) are considered a core component or building block of health systems. HISs are expected to support evidence-informed decision making at each level.^1^ However, there are two implicit, and commonly held assumptions that are important to challenge: first, that information systems require information technology; and second, that information technology has no place for paper.

While information systems are typically expected to involve the use of technology, the distinction between ‘information need’, and the technology to support the need, is important to consider especially (but not only) in low/middle-income country (LMIC) health system contexts with diverse constraints to technology implementation and use. Not every ‘information need’ requires the use of information technology, and many goals like quality improvement (QI) of health services may be achievable without the additional complexity of technology implementation.^2 3^

## Going ‘paperless’ is hard

When the decision to use information technology is made, ‘going digital’ is commonly equated with ‘going paperless’.^4 5^ Going paperless is challenging, especially for healthcare delivery in resource limited settings.^6^ Two important hurdles to digital (including mobile) health in LMICs stand out: (a) costs and complexities around infrastructure (not only of devices like computers/tablets/phones, but also backup power systems, networking, support, maintenance and procurement), and (b) costs and complexities around training (of diverse health system actors, often repeatedly, on complex hardware, software and workflows). Beyond these challenges, the shift to direct digital data entry at the point of care has a negative impact on both patient and provider satisfaction with the interaction. These are under-valued aspects of quality beyond technical skill, as time taken for direct digital data entry replaces time for direct patient–provider conversation and aspects like eye contact and non-verbal observation.^7^

## Paper as an interface for information entry

Paper, however, continues to be a simple, versatile, accessible and commonly used ‘interface’ for clinical documentation. Paper has few of the infrastructural and training challenges linked to digital technologies, and has advantages including automatic hard copies that may allow sites to meet legal requirements for documentation retention and patient privacy more easily. It also is preferred by many clinicians to document direct patient consultations. However, use of paper-based information is time consuming and expensive, either requiring direct reading or transcription/extraction into computer systems that involves numerous steps and yields low data quality.^8^ These have been the drivers for the move towards direct digital data entry, and the rapid proliferation and use of mobile devices have supported an assumption that paper-based documentation is incompatible with modern information systems.^9 10^ However, in settings where infrastructure, resources and skills are constrained, low-tech approaches may still be best for certain tasks. We are making the case for more thoughtful, goal-directed use of paper in combination with available digital tools and user abilities.

## Paper + digital

**Figure 1:**
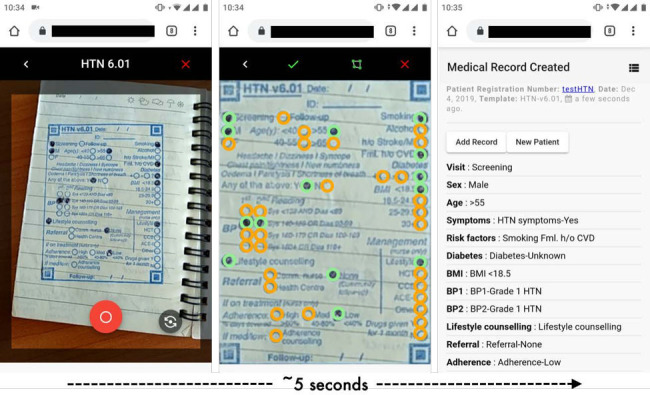
‘Paper-to-digital’ electronic medical records. Smartphone screenshots show how paper-based templates for clinical documentation, in this example for hypertension (HTN) screening by a community health worker, are combined with a browser-based computer vision application that automatically recognises the filled circles seconds after taking an image of the template. The interface allows for visual confirmation of accuracy and quick editing (if needed), followed by sync-ing of data to a cloud server.

One hybrid approach is to use the computational power of smartphones to automatically recognise information entered in paper. While handwriting recognition is still difficult to automate at accuracy levels needed for medical use,^11^ the ‘optical mark recognition’ (OMR) approach is trusted by anyone familiar with shading circles to answer multiple-choice questions. Recent work demonstrates the combination of paper-based clinical documentation templates containing OMR fields, with a computer vision algorithm on smartphones to automatically digitise patient records.^12^ In this case, templates are printed using rubber stamps, a widely available and low-cost solution for printing on demand, and the algorithm generates digital data from a smartphone picture of the template (figure 1). The approach has demonstrated improvements in both clinical documentation^13^ and care quality^12^ with minimal infrastructure or training. While useful for capturing structured data, the approach does not support capture of narrative information, or continuous variables like heart rate. Further innovation could provide solutions as experience with the hybrid approach grows.

## From information to quality

To achieve high-quality care, however, a culture of quality must be fostered throughout the healthcare system. ‘Information use’ is an important element of such a culture, and is often a challenge in LMIC settings.^14^ A culture of information acquisition and use is needed at all levels of the health system, in leaders—for setting goals and recognising performance, managers—for supervision and implementation and individual health workers in their own tasks of providing care. Underpinning such a culture shift will be the availability high-quality, routine data.^15^ The hybrid, paper-digital information ecosystem, as currently implemented in East Africa,^16^ allows managers to routinely track and respond to individual provider performance, as well as examine trends across a facility or district in a learning or quality improvement network. This capitalises on the advantages of paper for rapid documentation of patient consultations and those of digital data for tasks like quality improvement or referral management. A routine HIS using low cost, hybrid paper-digital approaches to information capture can improve equity of high-quality healthcare provision, ensuring that not only hospitals with the finances to afford expert clinical audits can support clinicians on the quality of services they provide,^17^ and deliver opportunities for individual and system improvement.^18^

## The art and science of measurement in information systems

While a routine HIS using a hybrid paper-digital approaches is likely to improve on what is currently being done around quality improvement in LMIC health systems, even such frugal innovations require investment. Therefore, evaluation of effectiveness and cost-effectiveness is required. Evaluation is challenging because of the inherent complexity of both the intervention and the health systems in which it is embedded. Any evaluation of the effectiveness or cost-effectiveness of routine HIS would require combining relatively straightforward process improvements (eg, comparing individual provider behaviour with those expected from clinical guidelines), with health outcomes in target clinical areas. However QI interventions like these often yields benefits in less tangible areas such as provider motivation, cohesion (team work), retention and resilience.^19^ These are more difficult to quantify and careful thought of study designs and novel approaches are likely needed to demonstrate the benefit of such interventions.^20^

## Paper-first, evidence-informed decision making

Interventions generally work best when it is not too radical a leap for patients, healthcare providers and managers to make, and when systems are designed to accommodate specific contexts and constraints in LMIC health systems. The ‘paper-first’ approach, by relying on existing resources and practices, is one step in this direction. A further innovative leap in the paper-digital approach is the capture of digital data by simply taking a picture using commonly available, even personal, mobile devices. Together—information entry on paper, and information capture by taking a picture—the paper-digital approach reduces several barriers to generating routine health information such as infrastructure, training, power and stable internet. But ultimately, it is likely the familiarity with ‘existing ways of doing things’ like entering notes on paper, or taking a picture on any mobile phone, that will make the paper-digital approach adopted at scale. The democratisation of information generation and use is likely to empower individuals and teams to drive change even in resource-constrained health systems.^21^

These approaches should have appeal at the national health ministry level, especially when they can be designed and tracked to provided critical information to support universal health coverage (UHC) efforts, or linked with existing HIS infrastructure, such as DHIS2.^22^ UHC planning and implementation are challenged by an underlying lack of actuarial data to support and assess system (re-)design. UHC Task Forces might be able to provide pilot funding to test novel, hybrid paper-digital hybrid approaches to meet their data needs. At the provider level, the appeal is a manageable level of staff training time and a seamless approach to activity that is the norm currently. There is investor appeal by virtue of low entry and implementation costs, and a dramatic improvement in information to guide governance and further investment decisions. Further innovation along these lines needs support in the global health community; the investment needed is typically small, low risk and potentially high value.

## Conclusion

It is imperative for the research, implementation and financing communities, both local and global, to embrace a concept of HISs that is not characterised as an inevitable, if slow, evolution from paper to digital but a thoughtful, context-sensitive, integration of the two.

## Data Availability

There are no data in this work.
